# Prospective Observational COVID-19 Screening and Monitoring of Asymptomatic Cancer Center Health-Care Workers with a Rapid Serological Test

**DOI:** 10.3390/diagnostics11060975

**Published:** 2021-05-28

**Authors:** Angelo Virgilio Paradiso, Simona De Summa, Nicola Silvestris, Stefania Tommasi, Antonio Tufaro, Angela Maria Vittoria Larocca, Vincenzo D’Addabbo, Donata Raffaele, Vito Cafagna, Vito Michele Garrisi, Giuseppe De Palma

**Affiliations:** 1 Scientific Direction, IRCCS Istituto Tumori “Giovanni Paolo II”, 70124 Bari, Italy; a.paradiso@oncologico.bari.it (A.V.P.); d.raffaele@oncologico.bari.it (D.R.); 2 Molecular Diagnostics and Pharmacogenetics Unit, IRCCS Istituto Tumori “Giovanni Paolo II”, 70124 Bari, Italy; s.tommasi@oncologico.bari.it; 3 Unit of Internal Medicine “Guido Baccelli”, Department of Biomedical Sciences and Human Oncology, University of Bari, 70124 Bari, Italy; nicola.silvestris@uniba.it; 4 Medical Oncology Unit, IRCCS Istituto Tumori “Giovanni Paolo II”, 70124 Bari, Italy; 5 Experimental Oncology and Biobank Management Unit, Institutional BioBank, IRCCS Istituto Tumori “Giovanni Paolo II”, 70124 Bari, Italy; a.tufaro@oncologico.bari.it (A.T.); g.depalma@oncologico.bari.it (G.D.P.); 6 Hygiene Unit, University Hospital “Policlinico” of Bari, 70124 Bari, Italy; laroccaangela1@gmail.com; 7 Professioni Sanitarie Unit, IRCCS Istituto Tumori “Giovanni Paolo II”, 70124 Bari, Italy; v.daddabbo@oncologico.bari.it; 8 Clinical Pathology Laboratory, IRCCS Istituto Tumori “Giovanni Paolo II”, 70124 Bari, Italy; cafagnavito83@libero.it (V.C.); vito.m.garrisi@oncologico.bari.it (V.M.G.)

**Keywords:** COVID-19, SARS-CoV-2, serological test, health-care workers, cancer center

## Abstract

Health-care workers (HCW) are at high risk for SARS-CoV-2 infection and, if asymptomatic, for transmitting the virus to fragile cancer patients. We monitored all asymptomatic HCWs of a cancer institute (94% of all employees agreed to enter the study) with the rapid serological test, VivaDiag^TM^, identifying SARS-CoV-2 associated-IgM/IgG. The tests were performed at time 0 (*n* = 606) and after 14 days (*n* = 393). Overall, the VivaDiag^TM^ results of nine HCWs (1.5%) were positive, with one confirmed to be SARS-CoV-2-positive after oropharyngeal swab testing by RT-PCR. At time 0, all nine cases showed IgM expression while IgG was detected in only one. After 14 days, IgM persisted in all the cases, while IgG became evident in four. A chemiluminescence immunoassay (CLIA) confirmed IgM positivity in 5/13 VivaDiag^TM^ positive cases and IgG positivity in 4/5 VivaDiag^TM^ positive cases. Our study suggests that the VivaDiag^TM^ test can be of help in identifying SARS-CoV-2 infected people in cohorts of subjects with a high prevalence.

## 1. Introduction

As of 25 April 2020, there were 3,073,603 coronavirus disease 2019 (COVID-19) cases (211,768 deaths, 1,937,184 active cases with 1,880,893 with mild disease and 56,291 with serious or critical disease) in the world [1]. The unfoldingof the pandemic has forced each of us to face a life-threatening situation, with health systems, even in the most advanced countries, severely challenged by the crisis [2].

Since the first cases were reported in China, we have learned that this infection can present with a heterogeneous clinical picture in terms of severity, ranging from completely asymptomatic to serious and potentially fatal forms [3]. While the latter represent a dramatic challenge, often requiring admission to intensive care units, undetected asymptomatic cases may drive the spread of the infection [4]. This becomes especially relevant for health-care workers (HCW) who are in contact with frail individuals with different diseases. In particular, cancer patients represent a risk category with significant management challenges due to both the risk of contagion and possible major complications related to the infection [5,6]. The incidence of COVID-19 has been reported to be 16% for HCWs of COVID-19 departments [7]. This figure is 4.1% for HCWs of wards with patients with respiratory symptoms without laboratory evidence of SARS-CoV-2 positivity [8]. Reports of antibody positivity in HCW cohorts early in the pandemic ranged widely across studies, hospitals, and geographic settings from 3.4–1.4% in a large Canadian tertiary care center [9], 3.2% in an oncology department of an Austrian university hospital [10], to just 1% in a Californian regional health-care delivery network [11]. To date, there are no published data indicating the incidence of asymptomatic positive COVID-19 HCWs employed at a center dedicated exclusively to the treatment of cancer patients.

RT-PCR testing of nasopharyngeal swabs is the standard method for diagnosing COVID-19 despite its sensitivity issues and the fact that it is not easily administered to the general population [12,13]. Rapid and easy to handle serological tests can detect IgM and IgG antibodies, which reveal a person’s recent or prior contact with the virus [14]. Immunological information concerning previous epidemics, based on or involving other Coronaviruses (SARS-CoV and MERS-CoV), is not yet available for the current SARS-CoV-2 epidemics [15].

With the aim to quickly identify potential SARS-CoV-2 positive asymptomatic subjects, we screened a cohort of HCWs at the IRCCS Istituto Tumori “Giovanni Paolo II” of Bari (National Cancer Care and Research Center), a COVID-19-free cancer center. As secondary end-point, we addressed the following issues: (1) the prevalence of SARS-CoV-2 immunoreaction; (2) the kinetics of IgM and IgG in HCWs at a 2-week interval; and (3) the comparison of rapid serological test results with those of RT-PCR and CLIA assays.

## 2. Materials and Methods

As of 26 March 2020, all HCWs employed at IRCCS Istituto Tumori “Giovanni Paolo II” of Bari (doctors, nurses, allied health professionals, administrators, laboratory scientists and others) were invited to take part in a prospective trial to monitor SARS-CoV-2 IgM and IgG in venous blood by a rapid serological test undergoing assessment for WHO Emergency Use Listing, VivaDiag^TM^, which was previously validated by us [13]. Only asymptomatic HCWs were enrolled, defined initially as absence of cough, fever, dyspnoea, acute onset of anosmia, ageusia or dysgeusia. Blood samples were taken upon entering the study and then again after 2 weeks. The study protocol included standard RT-PCR testing of oropharyngeal swabs from individuals whose VivaDiag^TM^ test IgM or IgG results were positive. In a subset of cases, the VivaDiag^TM^ results and those of the IgM and IgG analysis by Chemiluminescence Assay (CLIA) Maglumi 800 were also compared. The study was approved by the Ethical Committee of the IRCCS Istituto Tumori “Giovanni Paolo II” of Bari, document number CE 872/2020.

A total of 606 HCWs (94% of the entire number employed at the cancer center) entered the first round of the study, while 393 of them (65%) also completed the second round (with the second blood sample) by the time the study ended (17 April 2020). After providing written informed consent, all participants filled out a questionnaire which collected information on the presence of clinical symptoms and the HCW’s possible risk of COVID-19 infection (contact with confirmed SARS-CoV-2 positive individuals or visits to areas with active SARS-CoV-2 circulation). Venous blood samples were then collected and immediately sent to the Clinical Pathology Laboratory (UNI EN ISO 9001:2015-certified) and to the BSL-2 laboratories of the Institutional Biobank (UNI EN ISO 9001:2015-certified) of the IRCCS Istituto Tumori “Giovanni Paolo II” of Bari for the VivaDiag^TM^ test.

The characteristics of the HCWs who entered the study are reported in Table 1. The median age was 47.5 years (range 20–73 years) and 39.4% were male. A total of 54.1% of the enrolled HCWs were involved in direct clinical activities, 9% in laboratory practice, 8% in administrative activities, and 28.9% in maintenance and cleaning activities. Of these, 1.1% reported minor clinical symptoms not directly related to COVID-19 disease, while 11.7% reported having had direct contact with individuals with suspected COVID-19 disease over the preceding two weeks. Six-point seven percent of the subjects had returned to work at the hospital after a quarantine period (none with a previous SARS-CoV-2 positive RT-PCR test). The clinical characteristics of the HCWs that completed the second part of the study were not statistically different from those of the whole starting series.

### 2.1. SARS-CoV-2 Rapid IgM/IgG Test

VivaDiag™ SARS-CoV-2 IgM/IgG Rapid Test combined antibody test kit (VivaChek Biotech, Hangzhou, China) is based on a lateral flow qualitative immunoassay for the rapid determination of the presence or absence of both anti-SARS-CoV-2-IgM and anti- SARS-CoV-2-IgG in human specimens (whole blood, serum, and plasma). A surface antigen from SARS-CoV-2, which can specifically bind to SARS-CoV-2 antibodies (including both IgM and IgG), is conjugated to colloidal gold nanoparticles and sprayed onto conjugate pads. The presence of SARS-CoV-2 IgG and IgM antibodies is indicated by a red or purple line that appears in the specific region for those antibodies on the device. Colorimetric reactions for IgM and IgG were separately evaluated and classified as negative, with weak or strong colorimetric reaction. Each test was evaluated by two operators and a picture of the colorimetric result was taken. In case of a disagreement between the two operators, the picture was evaluated by a third party.

### 2.2. Molecular Detection of SARS-CoV-2

In cases where the VivaDiag™ test was positive for IgM or IgG (*n* = 9), oropharyngeal swabs were collected the following day for standard SARS-CoV-2 RT-PCR testing. The RT-PCR tests were immediately performed at the Laboratory of Molecular Epidemiology and Public Health (Head: M. Chironna) of the University of Bari. Nucleic acid was extracted from swabs by MagNA Pure (Roche Diagnostics, Mannheim, Germany), according to the manufacturer’s instructions. The presence of the E gene, RdRP gene and N gene of the SARS-CoV-2 virus was identified by a commercial real-time PCR assay (Allplex 2019-nCoV Assay; Seegene, Seoul, Korea). Samples were considered positive if all three genes were molecularly detected. The CDC Real-time-PCR protocol was used to confirm the presence of SARS-CoV-2 [16].

### 2.3. Chemiluminescence (CLIA) IgM/IgG Detection

The IgM and IgG dosage were determined utilizing Maglumi 800 2019-nCoV IgM (Cat. 130219016M) and IgG (130219015M) (CLIA) according to the manufacturer’s indications (Shenzhen New Industries Biomedical Engineering Co., Ltd. (Snibe), Shenzhen, China). The kit was designed for indirect chemiluminescence qualitative-semiquantitative in vitro immunoassay of IgM and IgG antibodies against SARS-CoV-2 in human blood. The pre-diluted biological sample, buffer and magnetic microbeads coated with recombinant SARS-CoV-2 antigen were mixed and incubated to create immunocomplexes. After magnetic field exposure, IgM and IgG antibodies labeled with ABEI were added; the chemiluminescence starter activated the light reaction, which was detected by the photomultiplier Maglumi 800. The results were expressed as relative light units (RLU) and considered positive if the signal/cutoff (S/C) ratio was ≥1.

### 2.4. Statistical Analyses

Ninety-four percent of all the HCWs employed in our cancer center were recruited and 65% of them completed the study with the second round. The group of HCWs taking part in first round did not differ from the group that went on to complete the second round in terms of main clinical characteristics. Descriptive statistics were calculated, and when relevant, a two proportion z-test used. Statistical analyses were performed using R-software (Version 3.1.3). All data are available upon request.

## 3. Results

### 3.1. First Round

Ninety-four percent of all the HCWs employed at the National Cancer Research Center, IRCCS Istituto Tumori “Giovanni Paolo II” of Bari, were enrolled. Approximately 92% of the participants had routine daily contacts with clinical departments.

In 7/606 (1.1%) subjects, the VivaDiag^TM^ (Table 2) results were positive (weak or strong staining). IgM staining appeared in all 7 cases (5 weak and 2 strong) and in one the test showed a strong simultaneous IgM and IgG color reaction. Three cases, including the aforementioned, were HCWs who had had recent contact with COVID-19 patients. Of these, none presented clinical symptoms associated with COVID-19 disease. Only one was younger than 55 years of age. The day after the VivaDiag^TM^ test, oropharyngeal swabs were collected from all 7 subjects for SARS-CoV-2 RT-PCR testing. None tested positive for the virus (Table 2).

In order to gain further insights into the IgM and IgG analytical sensitivity provided by the colorimetric VivaDiag^TM^ kit, a blood plasma aliquot of all seven subjects was utilized for CLIA IgM and IgG analysis (Table 2). The CLIA showed IgM positivity in three cases (two nurses and a member of the hospital’s cleaning staff) and confirmed strong IgM and IgG positivity in one of them.

### 3.2. Second Round

In 7/393 (1.8%) subjects, VivaDiag^TM^ (Table 2) results were positive (weak or strong staining). IgM staining appeared in six cases and IgG color reaction in four. In one case, a strong reaction appeared simultaneously for IgM and IgG. The day after the VivaDiag^TM^ test, oropharyngeal swabs were collected again from all seven subjects for SARS-CoV-2 RT-PCR testing. One asymptomatic HCW without previous contact with high-risk subjects and with a weak colorimetric reaction for IgM and IgG, tested positive for SARS-CoV-2 (Table 2). The same individual also had positive CLIA results for IgG expression.

### 3.3. Monitoring Immunoglobulins

The availability of blood samples at an interval of 2 weeks highlighted variations in immunoglobulin levels over time. In brief, a shift towards IgG positivity was observed after two weeks in 4/9 subjects. Finally, two cases that were initially negative showed a weak positivity for both immunoglobulins at 14 days.

### 3.4. Interassay Comparison

All subjects with a positive VivaDiag^TM^ test result underwent oropharyngeal swabbing for SARS-CoV-2 RT-PCR testing. A 31-year-old, asymptomatic man employed as a cleaner, with a weak VivaDiag^TM^ IgM and IgG positivity, had positive RT-PCR results. When VivaDiag^TM^ and CLIA assays were compared, CLIA showed IgM or IgG positivity in 4/13 cases and VivaDiag^TM^ in 4/5 cases.

## 4. Discussion

Our study was based on the evidence that nosocomial transmission is a well-known amplifier of transmission in epidemics. Our three-pronged aim was to safeguard the health of our workers, permit rapid identification and isolation of infected HCWs, and protect patients from potential HCW-mediated cross-infection.

We therefore verified whether it was possible to screen asymptomatic HCWs using a serological test that identifies the presence or absence of anti-SARS-CoV-2 IgM and IgG. This study was designed to monitor the kinetics of immunoglobulin response to possible SARS-CoV-2 contact and to confirm the results of the colorimetric serological test used with respect to those provided by standard RT-PCR testing of oropharyngeal swabs and CLIA serological testing.

Our first observation concerns the prevalence of serological test positivity in our asymptomatic HCWs. In another setting, determination SARS-CoV-2 status of HCWs in COVID-19 hospitals yielded results that recently prompted the NHS to roll out HCV screening programs [17]. In our study, specifically concerning HCWs, 1.3% among the tested HCWs with positive serological results were RT-PCR negative for SARS-CoV-2. As expected, this prevalence value was significantly lower than the one reported in symptomatic HCWs from Wisconsin [7] and in those working in COVID-19 hospitals [8]. Although other experiences have reported five dozen oncology health-care professionals in a tertiary care hospital [10], to the best of our knowledge, this is the only study considering asymptomatic HCWs from a non-COVID-19 cancer center where a recently activated screening program for all hospitalized patients showed that 1% were SARS-CoV-2 positive (all promptly transferred to specific COVID-19 hospitals). The prevalence of Ig positive subjects increased to 1.8% in the second round of serological tests performed after a 14-day interval. Interestingly, one individual (completely asymptomatic) subsequently had positive RT-PCR results for SARS-CoV-2 infection.

Another point of our study concerned the kinetics of immunoglobulins assayed through a serological test in 393 subjects who had two blood samples taken at a 2 week interval. In general, an increase in IgG positivity was observed in the second samples (from 1 to 4 positive cases), in line with the common view describing IgM as responsible for early and IgG for late antibody reaction [15]. In a previous study [13], we compared serological test results with those of RT-PCR testing to confirm SARS-CoV-2 infection, and identified that IgM appeared at an early stage after infection. Recent data from Guo et al. [15] stressed the variability of Ig kinetics in SARS-CoV-2 patients and showed that COVID-specific IgG can be found upon onset of symptoms, while IgM can persist at high levels up to 25 days after symptom appearance. Our study on the use of a colorimetric qualitative serological test was not designed to acquire quantitative information on Ig kinetics, but it demonstrated that the serological test could help identify asymptomatic subjects who may have been exposed to SARS-CoV-2.

Finally, regarding the comparison of VivaDiag^TM^ results with those of molecular RT-PCR testing and CLIA, it is noteworthy that VivaDiag^TM^ detected an asymptomatic HCW who was subsequently confirmed to be SARS-CoV-2-positive after oropharyngeal swab testing by RT-PCR. As important as this finding was, approximately one thousand samples (first and second round) were screened to find that one positive HCW. Interestingly, the purpose of serological tests was not to find SARS-CoV-2 positive subjects, but rather to identify those immunized due to previous contact with the virus [18]. VivaDiag^TM^ was compared to qualitative-semiquantitative CLIA analysis of IgM and IgG in a total of 41 HCWs: 23 paired comparisons on HCWs that had returned to work after quarantining and had tested negative with both assays, while the remaining 18 had tested positive with VivaDiag^TM^. CLIA confirmed IgM and IgG positivity in only 7 out of the 18 positive VivaDiag^TM^ cases, while it confirmed IgG positivity in 4/5 positive VivaDiag^TM^ cases. These results seem to point to the different performances of the two tests. CLIA appears to be less sensitive in assaying IgM and IgG presence. However, all the HCWs with positive VivaDiag^TM^ results were asymptomatic before and after their blood was sampled, which suggests that the VivaDiag^TM^ results were false positives. That false positive tests may undermine the utility of COVID-19 serology testing has been stressed and associated with the low prevalence of COVID-19 infection in an asymptomatic population [19]. In fact, prevalence is the key factor affecting the positive predictive value of any diagnostic test [20].

In spite of the important aspect related with the involvement of an entire cancer center HCW population, our study suffers some limitations: the most delicate aspect concerns the unavailability of specificity performances of the utilized test. However, this aspect was previously analyzed by us [13]. Moreover, the comparison of VivaDiag^TM^ and CLIA test results were performed on a small group of subjects, thus needing to be further investigated in larger cohorts.

Serological monitoring of immunological response to vaccines in our HCW cohort is ongoing, with first data planned to be analyzed within the year. On the contrary, to the best of our knowledge, no specific information concerning the utilization of rapid serological testing to monitor vaccinated subjects is available. On the other hand, a recent study conducted on vaccinated HCWs, via the use of a qualitative-semiquantitative electrochemiluminescence serological test, showed that COVID-19 positive HCWs had a single dose antibody titer ten times higher than that of naïve HCWs with a full vaccination schedule [21].

At time of the study, the practical outcome of this procedure resulted in a rapid pre-screening of all HCWs to be further addressed to RT-PCR COVID-19 diagnosis.

## 5. Conclusions

Our study suggests that VivaDiag^TM^ assay can be helpful in identifying SARS-CoV-2 infected individuals in cohorts of subjects with a high prevalence of infection. On the contrary, although our previous study highlighted that it may be useful for providing relevant information on people’s immunoreaction to COVID-19 exposure and, although there was a slight overlap in terms of results with CLIA, it was not suitable for antibody kinetics due to the qualitative nature of the results. Moreover, different performances of serological colorimetric and CLIA tools remain to be further analyzed in larger and specifically designed studies.

## Figures and Tables

**Table 1:** Characteristics of the cohort of health-care workers screened for SARS-CoV-2.

	First Round	Second Round
Cohort Characteristics	*n* = 606 (%)	*n* = 393 (%)
**Sex**		
Male	239 (39.4%)	142 (36.1%)
Female	367 (60.5%)	251 (63.9%)
**Age**	47.49 years (range: 20–73)	48.3 years (range: 20–66)
**Role**		
Clinical activity	328 (54.1%)	212 (53.9%)
Laboratory	54 (9%)	36 (9.1%)
Administrative	49 (8%)	79 (20.1%)
Maintenance and cleaning	175 (28.9%)	66 (16.8%)
**Subjects with SARS-CoV-2 contact**	71 (11.7%)	42 (10.7%)
**Subjects with minor symptoms**	7 (1.1%)	5 (1.2%)
**Quarantined subjects**	41 (6.7%)	23 (5.8%)

**Table 2:** Results of VivaDiag^TM^, RT-PCR and CLIA related to health-care workers with positive VivaDiag^TM^ results during the first round.

			First Round					Second Round				
ID	2019-nCoV Contacts	Minor Symptoms	VivaDiag^TM^ Test Result		SARS-CoV-2 RT-PCR	CLIA Analysis		VivaDiag^TM^ Test Result		SARS-CoV-2 RT-PCR	CLIA Analysis	
			IgM ^1^	IgG ^1^		IgM	IgG	IgM ^1^	IgG ^1^		IgM	IgG
						(AU/mL)	(AU/mL)				(AU/mL)	(AU/mL)
#1	No	No	Weak	Neg	Neg	1.715 *	0.172	Neg	Weak	Neg	0.294	0.152
#2	No	No	Neg	Neg	Neg	0.277	0.157	Weak	Weak	Neg	0.31	0.295
#3	Yes	No	Pos	Neg	Neg	1.130 *	0.132	Pos	Neg	Neg	0.546	0.294
#4	Yes	No	Neg	Neg	Neg	0.436	0.24	Weak	Weak	Pos	0.391	5.397 *
#5	Yes	No	Weak	Neg	Neg	0.492	0.39	Weak	Neg	Neg	0.274	0.108
#6	No	No	Weak	Neg	Neg	0.569	0.15	Neg	Neg	Neg	0.3	0.119
#7	No	No	Weak	Neg	Neg	0.826	0.283	Pos	Neg	Neg	0.296	0.08
#8	Yes	No	Pos	Pos	Neg	1.184 *	6.918 *	Pos	Pos	Neg	0.772	9.96 *
#9	No	No	Weak	Neg	Neg	0.365	2.611 *	Neg	Neg	Neg	-	-

* Cut-off for CLIA positivity > 1 AU/mL; ^1^ Weak: low-intensity band; Pos: positive result; Neg: negative result.

## Data Availability

The data presented in this study are available on request from the corresponding author. The data are not publicly available due to privacy.
